# Analysis of Tear Ferning Patterns in Young Female Subjects with Refractive Errors

**DOI:** 10.1155/2021/9524143

**Published:** 2021-01-23

**Authors:** Mana A. Alanazi, Gamal A. El-Hiti, Alaa Al-Madani, Raied Fagehi

**Affiliations:** ^1^Department of Optometry, College of Applied Medical Sciences, King Saud University, P.O. Box 10219, Riyadh 11433, Saudi Arabia; ^2^Cornea Research Chair, College of Applied Medical Sciences, King Saud University, P.O. Box 10219, Riyadh 11433, Saudi Arabia

## Abstract

To evaluate the dry eye symptoms and ocular tear film in young female subjects with refractive errors (RE) using the ocular surface disease index (OSDI), phenol red thread (PRT) and tear ferning (TF) tests. *Methods*. A group of 50 young female subjects (mean ± standard division = 20.3 ± 1.1 years) with RE (−0.25 to −6.00D) completed the study. An age-matched control group consisting of 50 healthy normal eye female subjects (22.2 ± 1.5 years) was recruited for comparison. The OSDI was completed first, followed by PRT and TF tests. *Results*. Median OSDI and TF scores were significantly higher (Mann–Whitney test; *P* < 0.001) among the study group subjects [median (interquartile range (IQR)) = 13.5 (15.3) and 1.6 (1.3), respectively] compared to the control group [6.0 (4.0) and 0.9 (0.8), respectively], whereas the median PRT score was significantly lower (Mann–Whitney test; *P*=0.003) in the study group [(27.5 (6.3) mm] compared to the control group [29.5 (5.0) mm]. For subjects within the mild RE group (*N* = 30), significant differences (Mann–Whitney test, *P* < 0.001 to 0.005) were found between the median OSDI, PRT, and TF scores and those recorded within the control group. For the subjects with moderate RE (*N* = 20), significant differences (Mann–Whitney test, *P* < 0.001 to 0.002) were found between the median OSDI and TF scores, and those recorded within the control group. *Conclusion*. The presence of RE in young females has a negative effect on tear film in terms of dry eye symptoms, tear volume, and TF grades. Dry eye symptoms experienced by subjects with RE and the TF grades were significantly higher compared with the control group. In addition, the tear volume was significantly lower in the study group. Clearly, RE has a risk factor for dry eye.

## 1. Introduction

Refractive errors (RE) are intrinsic eye disorders associated with several ocular problems [1]. RE can lead to defects within components of the ocular system because they prevent focusing of light on the retina [2]. Uncorrected RE are the main cause of serious ocular disturbances leading to visual impairment and blindness [3, 4]. It has been reported that uncorrected RE lead to visual impermanent in more than 100 million individuals and are responsible for approximately 7 million cases of blindness [5]. The loss in potential productivity due to RE has been estimated as at least 124 billion dollars [6]. Lack of awareness, early diagnosis, and treatment are common problems associated with RE disorders [7]. Hence, improving early RE detection and diagnosis is important for avoidance of visual complications. Visual impairment due to an uncorrected RE has a long-term negative effect on quality of life, social activity, education, employment opportunities, and health [8].

There are three main types of RE: myopia, hyperopia, and astigmatism [9]. Both myopia and hyperopia are caused by spherical errors, whereas astigmatism involves optical asymmetry [4]. Myopia (short-sightedness) occurs when the visual system has a high RE compared to the eyeball length [3] and is characterized by significant blurred vision when viewing distant objects [10]. Hyperopia (far-sightedness) occurs when the eye's axial length is too short compared to its optical power [4, 11]. In addition, hyperopia can occur due to low crystalline lens power or a relatively flat cornea [11]. Subjects having uncorrected hyperopia might experience conditions including blurred vision, strabismus, or binocular dysfunction [4]. In astigmatism, the visual system's RE differ across different meridians [12]. Astigmatism is considered a higher level of myopia or hyperopia [13], characterized by blurred vision for both distant and close objects [4]. RE can be corrected through the use of contact lens, glasses, or ocular surgery [9]. The eye's RE status provides valuable information about ocular health [14, 15]. For example, myopia can be associated with several ocular conditions such as glaucoma, optic atrophy, nystagmus, retinitis retinopathies, optic nerve hypoplasia, and toxoplasmosis [14]. Hyperopia has been linked to conditions including rod monochromacy, microphthalmia, and achromatopsia [14]. Astigmatism has been linked to albinism, chalazion, and pellucid marginal degeneration [14].

Various treatment procedures are available for the RE in which photorefractive keratectomy (PRK) is very common [3]. Such procedure involves the removal of the membrane of the epithelial basement followed by laser photoablation of the anterior stroma and Bowman's layer [16]. However, after the PRK, the concentration of free radical increases within the corneal tissues which could lead to alterations to both the structure and functional of stromal and epithelial corneal cells [17]. Interestingly, the use of oral eye supplements (*L*-cysteine and basic fibroblast growth factors eye drops) leads to a significant improvement in healing the corneal wound after the PRK in a relatively short time [16, 18].

Dry eye disease has become a major health concern since it is the most common ocular disorder [19, 20], associated with various symptoms of discomfort, ocular surface damage, and visual disturbances [20]. Dry eye has various causes but is mainly due to either insufficient tear secretion or a high rate of tear evaporation [20]. Dry eye increases with age and can affect any race or gender but is more common in females [21]. There is no single diagnostic procedure for detecting dry eye [22]. Symptoms of discomfort associated with dry eye can be detected using a questionnaire such as the ocular surface disease index (OSDI) [23]. The most common techniques for detecting dry eye and assessing the tear film include Schirmer [24], phenol red thread (PRT) [24], tear meniscus height (TMH) [25], noninvasive tear breakup time (NITBUT) [25], osmolarity [26], tear evaporation rate (TER) [27], and tear ferning (TF) [28] tests.

Various reports have discussed the association between dry eye and RE disorders based on measurements obtained from structural questionnaires and Schirmer, NITBUT, and TMH tests [29–33]. The current study is the first to analyze tear ferning patterns in young female subjects with RE using the TF test. The TF test has been used to assess tear quality previously, with proven repeatability [34–43]. In addition, the PRT test was used in the current work to measure aqueous content within the eye in the study participants.

## 2. Methods

### 2.1. Subjects

A group of 50 young female subjects aged 18–30 years (mean ± standard division 20.3 ± 1.1 years) with RE (−0.25 to −6.00D) completed the study. An age-matched control group consisting of 50 healthy normal eye female subjects (22.2 ± 1.5 years) was also recruited for comparison. The RE prescription of the enrolled subjects has been updated before the commencement of the study. The subjects were treated based on the Declaration of Helsinki [44]. Written informed consent was obtained from each subject before conducting the study. Subjects with thyroid disorders, a high cholesterol level (>4 mmol/L), a high body mass index (≥25 Kg/m^2^), vitamin A and D deficiencies, hypertension, anemia, or diabetes, and smokers, subjects with a history of ocular surgery, current or past contact lens wearers, and pregnant or breastfeeding individuals were excluded. The subjects were tested at the Clinics of the College of Applied Medical Sciences, Riyadh, between 09:00 and 11:00 am, by the same examiner. The environment was controlled in terms of temperature (20°C), humidity (25%), and rate of airflow. The OSDI was completed first, followed by the PRT and TF tests. The tests were carried out on the right eye of each subject and a 5-minute gap was allowed between tests [45].

### 2.2. The OSDI

The OSDI was completed and a normal eye was defined for a score less than 13 [46].

### 2.3. The PRT Test

Zone-Quick cotton threads were purchased from Showa Yakuhin Kako Co, Ltd. (Tokyo, Japan). While the eye was in the primary position, a 3 mm portion of the cotton thread was folded and inserted one-third of the distance from the temporal canthus of the lower eyelid [47]. The thread was removed after 15 seconds and the red-colored portion was measured. A measurement greater than 10 mm was defined as a normal eye [45].

### 2.4. The TF Test

A glass capillary tube (10 *μ*L) purchased from Merck (Schnelldorf, Germany) was used to collect a tear sample (1 *μ*L) from the lower meniscus of the right eye of each subject. The tear sample was dried for 10 minutes at 23°C and a humidity of less than 40%. An Olympus DP72 digital microscope (Tokyo, Japan) was used to observe and capture the TF images (magnification power of 20x). The TF patterns were graded based on the five-point TF grading scale and a grade less than 2 was defined for a normal eye [28].

### 2.5. Statistical Analysis

Microsoft Excel 2010 (Microsoft Office, Microsoft Corp., Redmond, WA, USA) was used to record the data. The data were analyzed using the Statistical Package for the Social Sciences software (SPSS; IBM Software, version 22, Armonk, NY, USA). A correlation coefficient, defined as being strong (0.50–1.00) or medium (0.30–0.49), was used to describe the correlation between parameters [48]. The data were not normally distributed (Kolmogorov–Smirnov test; *P* < 0.05) for the OSDI scores, or the PRT and TF tests between study and control group subjects. Therefore, the median (IQR) was used to represent the average scores.

## 3. Results

The median (IQR) scores for the OSDI and the PRT and TF measurements among study and control group subjects are shown in Table 1. Significant differences (Mann–Whitney test) were found between the scores collected from study and control group subjects. The median OSDI and TF scores were significantly higher (Mann–Whitney test; *P* < 0.001) among study group subjects [13.5 (15.3) and 1.6 (1.3), resp.] compared to the control group [6.0 (4.0) and 0.9 (0.8), resp.]. Meanwhile, the median PRT score was significantly lower (Mann–Whitney test; *P*=0.003) among the study group subjects [(27.5 (6.3)) mm] compared to the control group [29.5 (5.0) mm].

The OSDI scores indicated that 48% of study group subjects (*N* = 24) experienced symptoms of dry eye. Based on the TF grades recorded in the study group, 18 subjects (36%) had a dry eye (TF grade ≥2). None of the study group subjects had a dry eye based on the measurements obtained from the PRT test, since the aqueous content within the eye was at a normal level (≥15 mm) in all cases. Clearly, RE is a risk factor for dry eye based on OSDI and TF scores.

**Figure 1:**
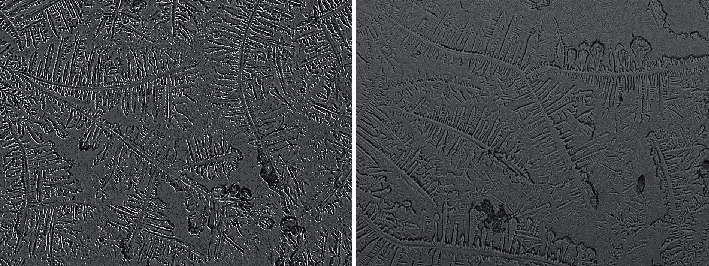
Representative TF images for two study group subjects.

**Figure 2:**
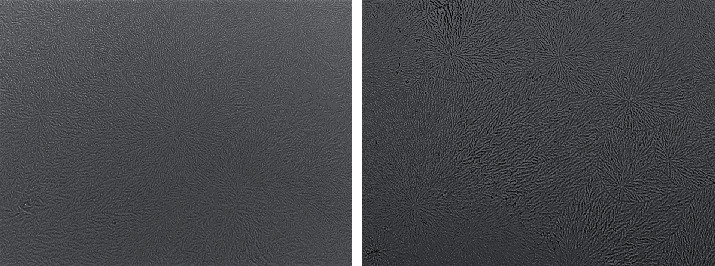
Representative TF images for two control group subjects.

**Figure 3:**
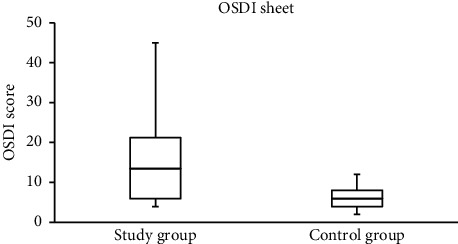
Side-by-side boxplots for the OSDI scores. A statistically significant difference at *P* < 0.001 (Mann–Whitney test).

**Figure 4:**
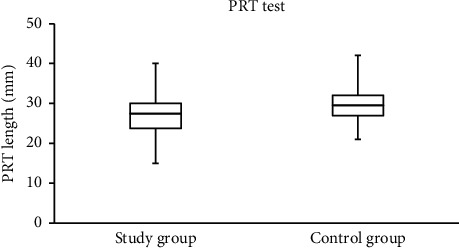
Side-by-side boxplots for the PRT scores. A statistically significant difference was found at *P*=0.003 (Mann–Whitney test).

**Figure 5:**
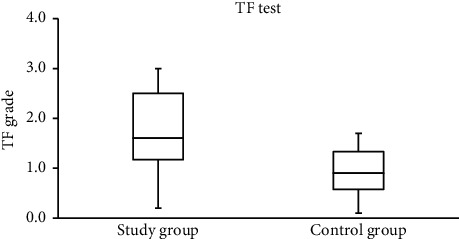
Side-by-side boxplots for the TF scores. A statistically significant difference was found at *P* < 0.001 (Mann–Whitney test).

Representative TF images obtained from two subjects from the study and control groups are shown in Figures 1 and 2, respectively. The side-by-side boxplots for the scores obtained from the OSDI, PRT, and TF tests among the subjects within both the study and control groups are shown in Figures 3–5, respectively.

The study group (*N* = 50) was subdivided into two categories based on the degree of REs, defined as mild (*N* = 30; –0.25 to –3.00D) and moderate (*N* = 20; −3.10 to −6.00D). The median OSDI, PRT, and TF scores for subjects within the mild and moderate RE groups are shown in Table 2. For subjects with mild RE (*N* = 30), significant differences (Mann–Whitney test, *P* < 0.001 to 0.005) were found between median OSDI, PRT, and TF scores and those recorded within the control group. For the subjects with moderate RE (*N* = 20), significant differences (Mann–Whitney test, *P* < 0.001 to 0.002) were found between median OSDI and TF scores and those of the control group. No significant differences (Mann–Whitney test, *P*=0.089) were found between the PRT measurements recorded among subjects with moderate RE and the control group. For subjects with moderate REs, there was a medium negative correlation (*r* = −0.463; *P*=0.040) between the TF grade and the OSDI score. It seems that the degree of RE has no effect on the severity of dry eye, based on the OSDI, PRT, and TF scores.

## 4. Discussion

Dry eye is a complex ocular disorder affecting a large percentage of the global population. The condition has been associated with aging [49], smoking [36], diabetes [37], thyroid gland disorders [38], and vitamin A and D deficiencies [41, 43]. In addition, it is more prevalent among females [21], possibly due to tear film disturbances resulting from changes in sex hormones [50]. Relatively few reports have suggested RE as a risk factor for dry eye [29–33, 51, 52]. Although dry eye is not common among young adults, it could be affected by daily activities such as prolonged use of computers, smartphones, or other digital devices. Therefore, the current study concentrated on investigating the association between dry eye and RE using the TF and PRT tests in young female subjects.

The current study suggests an association between dry eye and REs, based on the OSDI score and the measurements obtained from both the PRT and TF tests. The degree of RE (mild or moderate) does not affect the severity of dry eye. OSDI and TF median scores were significantly higher among the study group compared to normal eye subjects, whereas PRT median scores were significantly lower among RE subjects, although readings showed normal eye aqueous content for all subjects. These results are consistent with those previously reported. For example, the NITBUT readings obtained within a group of Saudi young females (*N* = 126) were found to be significantly lower among subjects with myopia and hyperopia compared with those recorded in a normal eye group [30]. However, the TMH readings recorded among myopic and hyperopic subjects showed normal eye readings [30]. The prevalence of dry eye among myopic, emmetropic, and hypermetropic subjects was 36.5, 24.6, and 17.4%, respectively [30]. Myopic teenagers (12.3 ± 11.9 years) have a high dry eye prevalence based on the OSDI, corneal fluorescein, and NITBUT scores [51]. The tear volume measured in young adults using Schirmer test (18–28 years; *N* = 90) indicated that dry eye was prevalent in hypermetropic (26.6%) and myopic (1.1%) subjects [31]. However, the average scores for tear volume in both hypermetropic (13.2 ± 5.0 mm) and myopic (18.4 ± 4.3 mm) subjects were at the normal level [31].

Based on a structural questionnaire, dry eye symptoms were detected in myopic (11.1%), hypermetropic (8.0%), and astigmatic (15.3%) subjects [32]. Dry eye was found to be more prevalent in female versus male subjects [32]. In addition, burning eye symptoms were common among hypermetropic subjects (18.7%) compared to subjects with myopia (12.5%) and astigmatism (12.5%) [32]. A survey based on dry eye and light sensitivity equations in a large population (*N* = 893) showed that dry eye was more prevalent among contact lens wears (53.2%) and spectacle wears (23.9%) compared to emmetropes (7.1%) [33].

An association between dry eye and uncorrected RE in females has been reported [53]. Tear breakup time was found to be associated with meibomian gland disorder [54]. Subjects with RE are among the most vulnerable to developing dry eye due to their frequent use of glasses and contact lenses [33]. The development of dry eye in individuals with RE could result from changes within the anterior surface of the cornea when the eyeball elongates [52, 55]. However, the exact mechanism associating dry eye and RE has yet to be elucidated.

## 5. Conclusion

Refractive errors among young female have a negative effect on the tear film in terms of dry eye symptoms, tear volume, and TF grades. Dry eye symptoms experienced by subjects with RE and the TF grades were significantly higher compared with the control group. In addition, the tear volume was significantly lower in the study group. Clearly, RE has a risk factor for dry eye. However, the degree of refractive errors has no effect on the severity of dry eye.

## Figures and Tables

**Table 1:** Median (IQR) scores for the OSDI, PRT, and TF tests for subjects in the study (*N* = 50) and control (*N* = 50) groups.

Parameter	Study group (*N* = 50)	Control group (*N* = 50)	*P* value
OSDI	13.5 (15.3)	6.0 (4.0)	<0.001
PRT (mm)	27.5 (6.3)	29.5 (5.0)	0.003
TF	1.6 (1.3)	0.9 (0.8)	<0.001

**Table 2:** Median (IQR) scores for the OSDI, PRT, and TF tests for subjects with mild (*N* = 30) and moderate (*N* = 20) RE.

Parameter	Mild RE (*N* = 30)	*P* value	Moderate RE (*N* = 20)	*P* value
OSDI	13.5 (21.5)	0.005	13.5 (13.5)	<0.001
PRT (mm)	26.5 (8.0)	0.002	29.0 (5.0)	0.089
TF	1.7 (1.3)	<0.001	1.5 (1.4)	0.002

## Data Availability

The data used to support the findings of this study are available from the corresponding author upon request.
